# Commentary: PFKFB3 overexpression in monocytes of patients with colon but not rectal cancer programs pro-tumor macrophages and is indicative for higher risk of tumor relapse

**DOI:** 10.3389/fimmu.2023.1290459

**Published:** 2023-12-20

**Authors:** Nikolay Shakhpazyan, Liudmila Mikhaleva, Arcady Bedzhanyan, Nikolay Sadykhov, Konstantin Midiber, Alexander Orekhov

**Affiliations:** ^1^Avtsyn Research Institute of Human Morphology, Petrovsky National Research Center of Surgery, Moscow, Russia; ^2^Department of Abdominal Surgery and Oncology II (Coloproctology and Uro-Gynecology), Petrovsky National Research Center of Surgery, Moscow, Russia; ^3^Institute of Medicine, Peoples’ Friendship University of Russia named after Patrice Lumumba, Moscow, Russia; ^4^Laboratory of Angiopathology, Institute of General Pathology and Pathophysiology, Moscow, Russia; ^5^Institute for Atherosclerosis Research, Moscow, Russia

**Keywords:** colorectal cancer, inflammation, metabolism, monocytes, cytokines

## 1 Introduction

The evolution and progression of colon cancer, akin to other solid neoplasms, constitute an elaborate nexus of multifarious biological interactions and dependencies. A notable exemplar is metabolic reprogramming, characterized by enhanced glycolytic activity coupled with diminished oxidative phosphorylation. Larionova et al. have convincingly illustrated that monocytes, isolated from the peripheral blood of patients with colon cancer, demonstrate pronounced upregulation of 6-phosphofructo-2-kinase/fructose-2,6-biphosphatase 3 (PFKFB3), a key enzyme in the glycolytic cascade (1). In our assessment, this seminal work paves the way for extensive future research into immunogenesis and inflammation in colon cancer.

## 2 The new perspectives on the study of metabolic reprogramming of monocytes in colorectal cancer

In line with recent findings by Larionova et al., which highlighted the marked upregulation of the glycolytic enzyme PFKFB3 in circulating monocytes derived from colorectal cancer patients, our study serves to augment and elaborate upon the current scientific understanding of these metabolic abnormalities. Larionova et al. posited that dysregulated monocyte metabolism plays a significant role in the pathogenesis of colorectal cancer. They further hypothesized a potential correlation between elevated PFKFB3 expression and altered immune functions, substantiated by a transcriptomic analysis that revealed perturbations in several genes, notably those governing the functions of TNF-α and IL-1β cytokines.

These results make us look at our study from a new perspective. Our research was conducted on a more limited cohort, comprising 12 patients diagnosed with colorectal cancer and 9 healthy controls. Employing enzyme-linked immunosorbent assays (ELISA) in cultured monocytes, we identified aberrant secretion patterns of pro-inflammatory cytokines. Specifically, upon a 24-hour stimulation with lipopolysaccharide (LPS), monocytes from colorectal cancer patients displayed elevated secretion levels of TNF-α and IL-1β in the culture medium. Intriguingly, when LPS stimulation was repeated after 7 days, there was a marked reduction in TNF-α secretion compared to the healthy controls, while IL-1β secretion remained consistent (2). Our observations are consistent with previously published data (3, 4).

The divergence in the secretion profiles of TNF-α and IL-1β, regulated predominantly through the NF-κB and NLRP3 inflammasome pathways respectively, suggests an underlying mechanistic dichotomy that merits further investigation. The reduction in TNF-α secretion upon repeated stimulation could indicate an altered metabolic state in monocytes, consistent with the metabolic paradigms proposed by Larionova et al., particularly concerning glycolytic pathways modulated by PFKFB3.

## 3 Discussion

Our data, in synergy with the seminal work of Larionova et al., provide a robust foundation for further, large-scale investigations. Notably, future research could advantageously employ dual LPS stimulation protocols to emulate chronic inflammatory conditions, a pivotal determinant in colorectal cancer pathogenesis. The application of advanced immunological and proteomic methodologies, as contrasted with transcriptomic techniques, is poised to yield a more nuanced depiction of cellular cytokine secretion patterns, thereby contributing to a more comprehensive understanding of immune responses in colorectal cancer.

**Figure 1:**
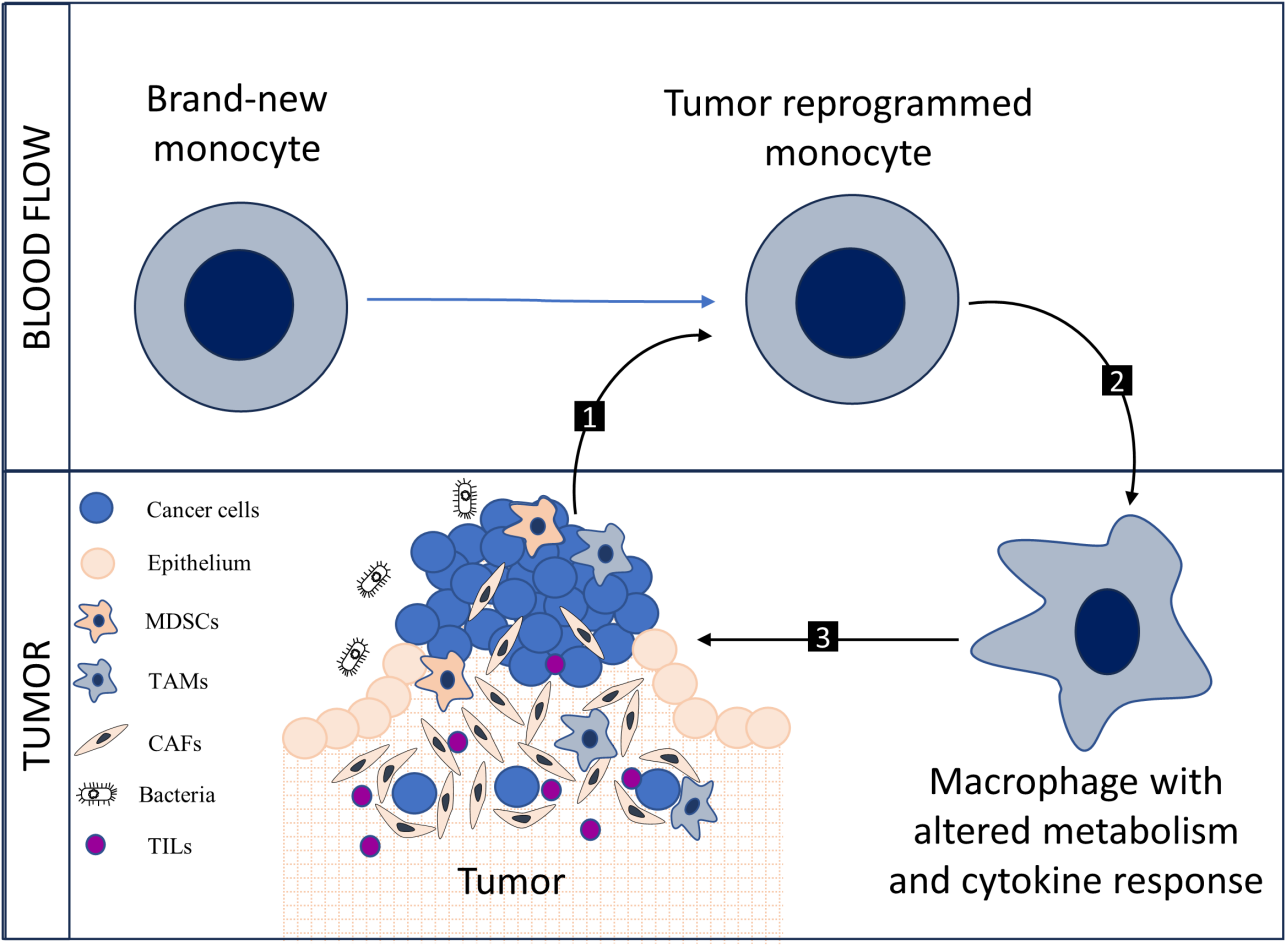
New monocytes undergo metabolic reprogramming under the influence of the tumor {1}, the shift towards glycolytic metabolism alters the cytokine potential of monocytes; monocytes transform into tumor-associated macrophages (TAMs) with modified metabolism and cytokine response {2}, a pro-tumoral interaction occurs between TAMs and components of the tumor stroma, including myeloid-derived suppressor cells (MDSCs), cancer-associated fibroblasts (CAFs), and tumor-infiltrating lymphocytes (TILs) {3}. Collectively, 1, 2, and 3 form a pathological cycle.

The metabolic alterations evident in circulating monocytes, specifically the notable upregulation of PFKFB3, establish an auspicious landscape for interdisciplinary research at the intersection of oncology, immunology, and cellular metabolism. Pertinent avenues for exploration include the phenotypic ramifications of PFKFB3 upregulation, the implications of metabolic exhaustion arising from sustained glycolytic activity, and the modulation of inflammatory signaling pathways by these metabolic shifts (5). Additionally, the overlap between metabolic reprogramming and epigenetic alterations offers another compelling domain of inquiry. Metabolic intermediates may act as cofactors for epigenetic enzymes, thereby exerting an influence on the transcriptional regulation of cytokines or other immune-related genes (6). In general, the formation of a pathological circle is possible (**Figure 1**).

We posit that future research in this domain would be significantly enriched by the adoption of dual LPS stimulation protocols, designed to emulate the chronic inflammatory conditions that are integral to the pathogenesis of colorectal cancer. Furthermore, we advocate for the employment of advanced methodologies spanning immunology, metabolomics, epigenetics, and proteomics. These approaches, we argue, could furnish a broader and more direct representation of cellular cytokine secretion compared to traditional transcriptomic techniques, thereby providing a comprehensive and dynamically nuanced portrait of immune responses implicated in this malignancy.

In summary, these diverse research trajectories not only corroborate the invaluable observations initially made by Larionova et al., but also underscore the complexity and multifactorial nature of colorectal cancer pathogenesis. Such avenues furnish a fertile ground for comprehensive studies employing proteomic, metabolomic, and *in vitro* cell culture methodologies, aimed at disentangling the convoluted mechanisms of immune regulation in colorectal cancer. This, in turn, may pave the way for the development of innovative therapeutic strategies.

## Author contributions

NSh: Conceptualization, Visualization, Writing – original draft. LM: Conceptualization, Supervision, Writing – review & editing. AB: Conceptualization, Supervision, Writing – review & editing. NSa: Funding acquisition, Writing – review & editing. KM: Validation, Writing – review & editing. AO: Conceptualization, Project administration, Supervision, Writing – review & editing.
